# Selected articles from the Third International Workshop on Semantics-Powered Data Analytics (SEPDA 2018)

**DOI:** 10.1186/s12911-019-0855-3

**Published:** 2019-08-08

**Authors:** Zhe He, Jiang Bian, Cui Tao, Rui Zhang

**Affiliations:** 1 School of Information, Florida State University, 142 Collegiate Loop, Tallahassee, FL 32306 USA; 2 Department of Health Outcomes and Biomedical Informatics, University of Florida, Gainesville, FL USA; 3 School of Biomedical Informatics, University of Texas Health Science Center at Houston, Houston, TX USA; 4 Institute for Health Informatics and College of Pharmacy, University of Minnesota, Minneapolis, MN USA

**Keywords:** Health data analytics, Ontology, Data mining, Semantic web

## Abstract

In this editorial, we first summarize the Third International Workshop on Semantics-Powered Data Analytics (SEPDA 2018) held on December 3, 2018 in conjunction with the 2018 IEEE International Conference on Bioinformatics and Biomedicine (BIBM 2018) in Madrid, Spain, and then briefly introduce five research articles included in this supplement issue, covering topics including Data Analytics, Data Visualization, Text Mining, and Ontology Evaluation.

## Background

With an increasing volume of electronic health data collected from various sources, the overlap between biomedical informatics and data science has been more significant than ever before [1]. Enabled by powerful machines and evolving machine/deep learning algorithms, unprecedented opportunities exist in harnessing the vast amount of health data for diagnosis, prognosis, outcome prediction, and personalized medicine. However, health care data are known to be heterogeneous and complex, posing significant challenges in the entire process of health data analytics from data acquisition, data preprocessing, data representation, analytics, to result visualization, presentation and interpretation. The reasons for the complexity of health data are multifaceted. Regarding the structured data, different health information systems may adopt different standard vocabularies or use customized local vocabularies to represent medical diagnoses, laboratory tests, medications, etc. In recent years, common data models such as the Observational Medical Outcomes Partnership (OMOP) Common Data Model along with its core vocabulary have been widely used in merging clinical data from heterogeneous sources for downstream analysis [2]. On the other hand, much valuable information is locked in free-text clinical narratives in patients’ electronic health records (EHRs) and postings on social media sites. However, extracting information from these free-text sources accurately and effectively is challenging as they often contain partial sentences, acronyms, and synonyms, requiring robust and tailored natural language processing (NLP) methods and tools.

The National Institute of Health (NIH) Data Science Strategic Plan released in June 2018 explicitly commits to ensuring that all data-science activities and products supported by the agency adhere to the FAIR principles, meaning that data be Findable, Accessible, Interoperable, and Reusable. In our view, ontologies and semantic web technologies will play a crucial role to address the FAIR principles. With the rapid growth of infrastructure support such as National Center for Biomedical Ontology (NCBO)'s BioPortal [3] for ontology catalog and maintenance and the Center for Expanded Data Annotation and Retrieval (CEDAR) workbench for metadata creation and validation [4], researchers have been empowered to use ontologies and semantic web technologies for knowledge representation, semantic annotation and inference, natural language processing, machine learning, and data analytics in general.

## SEPDA workshop

In the past three years since 2016, the International Workshop on Semantics-Powered Data Analytics (SEPDA) has brought researchers and practitioners in ontologies, data mining, knowledge representation, knowledge management, and data analytics to discuss innovative semantic methods, applications, and data analytics to address problems in healthcare, biomedicine, public health, and clinical research with biomedical, clinical, behavioral, and social web data. The submissions of SEPDA tackled critical problems in biomedical informatics such as extracting drug-drug interaction, drug repurposing, adverse drug reaction, detecting early signals for cognitive impairment, and visualizing dietary supplement knowledge. Gladly, we have seen a growing and diverse use of ontologies and semantic web technologies for health data analysis, linked open data, information extraction, knowledge base construction, and deep learning. We have published two journal issues for SEPDA 2016 [5] and SEPDA 2017 [6], respectively. SEPDA 2018 was held on December 3, 2018 in conjunction with the 2018 IEEE International Conference on Bioinformatics and Biomedicine (BIBM 2018) in Madrid, Spain.

## Summary of selected papers from SEPDA 2018

This supplement aims to showcase the state-of-the-art research and development efforts that effectively use biomedical ontologies and/or semantics methods to address important problems in health and biomedicine. The five selected papers from SEPDA 2018 underwent a rigorous review and revision process. We are glad to present selected papers that introduce novel tools, methods, and applications for Semantics-Based Data Analytics [7, 8], Data Visualization [9], Text Mining [10], and Ontology Evaluation [11]. We introduce them one by one as follows.

Venous thromboembolism is a preventable cause of death. Even though Padua linear model is a widely-used risk assessment method for venous thromboembolism, its utility in China is limited due to genetic and environmental differences between Western and Chinese population. In [7], Yang et al. built a risk prediction model for venous thromboembolism using the admission notes and progress notes in patients’ medical records. To obtain comprehensive information of patients, they used multiple biomedical ontologies including Medical Subject Headings (MeSH), Human Phenotype Ontology (HPO), SNOMED CT, and International Classification of Diseases Version 10 (ICD-10). Using rule-based section extraction, automatic ontology enrichment, and machine learning, they were able to identify salient medical terms that are effective in predicting venous thromboembolism with an optimal AUC of 0.97, which significantly outperformed the Padua model. This work shows the promise of using ontologies to facilitate information extraction from free-text data in EHRs for machine-learning-based prediction models.

The past decades have witnessed significant advancement in the development of antiviral agents for hepatitis C virus (HCV); and now the cure rate of HCV is more than 95%. Nevertheless, drug safety is always a concern, and post-market surveillance of adverse events (AEs) requires significant effort. Comparing to traditional venues of AE reporting such as the US Food and Drug Administration (FDA) Adverse Event Reporting System (FAERS), EHR systems is a new source of AE information. Huang et al. [8] introduced a statistical procedure to compare the difference in AEs across multiple HCV drugs using data from both FAERS and EHR, and to assess the consistency of results from the two data sources. Nevertheless, EHR systems are not designed for pharmacovigilance, so the events are recorded using the International Classification of Disease (ICD) codes rather than standardized terminology for AEs, posing challenges to accurately extract AEs from EHRs.

Dietary supplements (DSs) are widely used but consumers know little about their efficacy and safety. Online resources about such knowledge on DSs are scattered with various level of granularity. Researchers have developed a web application, ALOHA, to facilitate consumers’ needs on DS. In [9], researchers followed the user-centered design (UCD) principles, and carried out three design iterations to enrich the functionalities of ALOHA. The usability was evaluated with a modified system usability score increased compared with the original prototype. They demonstrated that graph-based interactive visualization is acceptable approach to end users who are interested in seeking online DS information.

The aging population has led to an increase in cognitive impairment (CI); however, it is unclear about the temporal trend of patient health functions and how they are related to the health needs. Using EHR data especially clinical texts on CI exploration has not been investigated. In [10], researchers examined temporal trends of patient activity of daily living and analyzed topics of patient medical conditions in EHR data in order to characterize and understand early signals of elderly patient CI. They have collected 1,435 CT patients and 1,435 CU patients from Mayo Clinic biobank. Using topic modeling on clinical text to examine patients’ medical condition change over time, they found that the temporal trends of basic and instrument ADL between CI and CU patient are significantly different. The trajectories of certain ADL were associated with CI patients.

Biomedical ontologies play an important role in knowledge representation, data integration, natural language processing, as well as decision support for health information systems and biomedical research. Ontology evaluation is an essential phase in ontology engineering. In [11], Amith et al. introduced OntoKeeper, a web-based application that permits ontologists to grade the quality of their ontology based on semiotic measures. The metric suite is composed of four branches - syntactic, semantic, pragmatic, and social. The syntactic score concerns the machine-readability of the ontology artifact, specifically asking if the ontology “can be read”. The semantic score assesses the appropriateness of the entities’ labels within the ontology, or if the ontology “can be understood”. The pragmatic score pertains to measuring the utility of the ontology, or if the ontology is “useful”. Finally, the social score measures the ontology status among the community of ontologies (i.e. “Can it be trusted?”). All four of these scores could be weighted to tailor the overall evaluation.

## Discussion and conclusions

In the past three years, SEDPA has been established as a key venue for disseminating novel methods, applications, and research results related to the use of semantic web technologies and ontologies on health data. The five selected papers included in this issue not only present novel applications and methods of semantic web technologies and ontologies but also demonstrate their values in addressing challenging health problems such as reporting drug adverse events in EHRs [8], visualizing dietary supplement information for normal consumers [9], and detecting early signals of cognitive impairments in the aging population [10]. We are looking forward to seeing the sustainable impact of these work. We also envision a rapid growth of semantics-powered data analytics in biomedical informatics and other fields. We hope to organize more such events to promote the use of semantic web technologies and ontologies to address real-world problems with big data.

## References

[1] Ohno-Machado L (2013). Data science and informatics: when it comes to biomedical data, is there a real distinction?. J Am Med Inform Assoc.

[2] Haberson A, Rinner C, Gall W (2019). Standardizing Austrians claims data using the OMOP common data model: a feasibility study. Studies in health technology and informatics.

[3] Noy N. F., Shah N. H., Whetzel P. L., Dai B., Dorf M., Griffith N., Jonquet C., Rubin D. L., Storey M.-A., Chute C. G., Musen M. A. (2009). BioPortal: ontologies and integrated data resources at the click of a mouse. Nucleic Acids Research.

[4] Gonçalves RS, O’Connor MJ, Martínez-Romero M, Egyedi AL, Willrett D, Graybeal J, Musen MA. The CEDAR workbench: an ontology-assisted environment for authoring metadata that describe scientific experiments. InInternational semantic web conference 2017 (pp. 103–110). Springer, Cham. 10.1007/978-3-319-68204-4_10 PMC7098808 32219223

[5] He Z, Tao C, Bian J, Dumontier M, Hogan WR (2017). Semantics-powered healthcare engineering and data analytics. Journal of Healthcare Engineering.

[6] He Z, Tao C, Bian J, Zhang R, Huang J (2018). Introduction: selected extended articles from the 2nd international workshop on semantics-powered data analytics (SEPDA 2017). BMC Med Inform Decis Mak..

[7] Yang Y, Wang X, Huang Y, Chen N, Shi J, Chen T. Ontology-based venous thromboembolism risk assessment model developing from medical records. BMC Med Inform Decis Mak. 2019. 10.1186/s12911-019-0856-2 10.1186/s12911-019-0856-2 PMC6686216 31391095

[8] Huang J, Zhang X, Tong J, Du J, Duan R, Yang L, Moore JH. Comparing drug safety of hepatitis C therapies using post-market data. BMC Med Inform Decis Mak. 2019. 10.1186/s12911-019-0860-6 10.1186/s12911-019-0860-6 PMC6686214 31391106

[9] He X, Zhang R, Rizvi R, Vasilakes J, Yang X, Guo Y, He Z, Prosperi M, Huo J, Alpert J, Bian J. ALOHA: developing an interactive graph-based visualization for dietary supplement knowledge graph through user-centered design. BMC Med Inform Decis Mak. 2019. 10.1186/s12911-019-0857-1 10.1186/s12911-019-0857-1 PMC6686235 31391091

[10] Goudarzvand S, Sauver JS, Mielke MM, Takahashi PY, Lee Y, Sohn S. Early temporal characteristics of elderly patient cognitive impairment in electronic health records. BMC Med Inform Decis Mak. 2019. 10.1186/s12911-019-0858-0 10.1186/s12911-019-0858-0 PMC6686236 31391041

[11] Amith M, Manion F, Liang C, Harris M, Wang D, He Y, Tao C. Architecture and usability of OntoKeeper, an ontology evaluation tool. BMC Med Inform Decis Mak. 2019. 10.1186/s12911-019-0859-z 10.1186/s12911-019-0859-z PMC6686219 31391056

